# Instruments for Measuring Healthcare Professionals’ Medication Safety Competence: A Scoping Review

**DOI:** 10.2147/JMDH.S508151

**Published:** 2025-03-25

**Authors:** Christoph Stephan, Doris Kloor, Viktoria Sophie Wurmbach, Cornelia Mahler

**Affiliations:** 1 Department of Nursing Science, Institute of Health Sciences, University Hospital Tuebingen, Tuebingen, Baden-Wuerttemberg, Germany; 2 Internal Medicine IX - Department of Clinical Pharmacology and Pharmacoepidemiology (Cooperation Unit Clinical Pharmacy), Medical Faculty Heidelberg/Heidelberg University Hospital (Heidelberg University), Heidelberg, Germany

**Keywords:** patient safety, competence assessment, nurses, physicians, pharmacists

## Abstract

Elderly patients with chronic diseases often use several drugs (polypharmacy). The more drugs a patient uses, the greater the risk of medication errors. To ensure a safe medication process, healthcare professionals must have a high level of medication safety competence (MSC). Hence, instruments are needed to measure healthcare professionals’ MSC and identify areas where improvement is required. A scoping review was conducted to obtain a comprehensive overview of existing instruments. The scoping review was carried out in accordance with the JBI methodology for scoping reviews and in line with the Preferred Reporting Items for Systematic Reviews and Meta-Analyses extension for Scoping Reviews (PRISMA-ScR). An a priori protocol was registered on Open Science Framework https://osf.io/73dmq. The search, performed in January 2024 in six databases, yielded 3740 results, duplicates were removed and 2499 titles and abstracts were screened. Full text screening of 28 articles resulted in the inclusion and analysis of 15 articles. A total of six instruments were identified, all instruments were designed for nurses or nursing students. However, no instrument to assess, eg physicians’ or pharmacists’ MSC was identified. Five of the six instruments were used in clinical setting and three in educational setting. The Medication Safety Competence Scale (MSCS) and the Medication Safety Scale cover the multidimensionality of MSC. The MSCS’s psychometric properties were considered acceptable. Thus, the MSCS can be used to assess nurses MSC in clinical and educational settings.

## Introduction

The older patients are, the more likely they are to have chronic diseases^1^ and, consequently, to use several drugs (polypharmacy).^2^,^3^ Polypharmacy, commonly defined as the use of more than five drugs,^4^ is associated with an increased risk of adverse health outcomes, such as frailty, hospitalizations and mortality^5–7^ and has also been linked to drug-related problems, such as inappropriate prescribing and (potential) medication errors (ME).^8–10^ Such ME can happen at every step of the complex medication process,^11^ and hence, medication safety is an essential component of patient safety.^12^ Medication safety is defined as:

Freedom from accidental injury during the course of medication use; activities to avoid, prevent, or correct adverse drug events which may result from the use of medications.^13^

Medication safety is an essential component of patient safety.^13^ Therefore, the World Health Organization (WHO) initiated the “Medication without harm” initiative as the third WHO Global Patient Safety Challenge. The goal was to reduce avoidable medication-related harm by 50% worldwide over a period of five years.^12^ But ensuring a safe medication process remains challenging due to the involvement of different people and different healthcare professionals within this process as well as the complexity of the different settings they are performed in and the medications themselves.^14^

Thus, all healthcare professionals involved in the medication process, eg physicians, nurses, pharmacists, have to be aware of the risks in the medication process and need to have the necessary competence to prevent ME and improve medication safety. Competence is hereby understood as a combination of knowledge, skills and attitudes^15^ that refers to the ability to perform a skill and the attribute of the performer, while the term “competency” is used for the “skill” itself.^16^ To date there is no agreed definition of Medication Safety Competence (MSC). The Safety Competencies Framework^17^ is a framework of multi-professional patient safety competencies which was originally developed by the Canadian Patient Safety Institute in collaboration with the Royal College of Physicians and Surgeons of Canada and revised in 2020. It comprises six different domains of patient safety competencies: Patient Safety Culture; Teamwork; Communication; Safety, Risk and Quality Improvement; Optimize Human and System Factors; Recognize, Respond to and Disclose Patient Safety Incidents. The concept of this framework was used and adapted for this review. Details are provided in the Methods section.

Medication safety competencies are primarily acquired in the various professional qualification programs, and different concepts have already been developed in order to teach them.^18–20^ In addition, there are already diverse international education programs to improve the competence of healthcare professionals in relation to (aspects of) MSC in order to increase medication safety in clinical practice.^21–23^

However, there is still a high prevalence of medication-related harm due to medication errors,^24^ with negative consequences for patients and high costs for healthcare systems, and in addition, it is not certain that HCPs’ (physicians, pharmacists and nurses) MSC is sufficient to reduce these harms in the future. Thus, assessment instruments to measure MSC are needed to determine whether healthcare professionals have the necessary MSC to ensure a safe medication process within an interprofessional team. It also allows for the identification of competence areas in which HCPs need improvement in order to target their development. This can have implications for professional qualification programs, for example, but also for continuing education activities. Therefore, following this review, a study is planned to evaluate and specifically improve the HCPs MSC.

Previous reviews have considered instruments for measuring patient safety competence^25^ or specific competencies, such as medication administration skills.^26^ However, the consideration of patient safety competence is too broad and general to make statements about the specific MSC required and to identify the need for action. Medication administration skills contribute to safety, but relate to the execution of techniques rather than the underlying competence. In addition, previous reviews and instruments often focus on nurses, whereas the interprofessional nature of the medication process requires consideration of all HCPs involved. It is therefore necessary to take a closer look at the specific field of MSC and to identify instruments that will enable all HCPs to be covered.

A search of MEDLINE, the Cochrane Database of Systematic Reviews, JBI Evidence Synthesis and Open Science Framework for existing systematic reviews, scoping reviews or protocols^27^ on instruments to measure MSC of healthcare professionals did not identify any relevant results. Thus, a scoping review which aimed to identify all relevant assessment instruments that measure the MSC of healthcare professionals was conducted. The review type was chosen as it is a good way of obtaining a comprehensive overview and presenting the current state of research.^28^ The review therefore offers the possibility of selecting an instrument for assessing the MSC. The assessment allows a structured analysis of the MSC in the different HCPs and the need for action at different levels can be derived.

## Review Question

Which instruments are available to assess healthcare professionals’ medication safety competence?

## Inclusion Criteria

### Participants

The scoping review focused on healthcare professionals, eg nurses, physicians, pharmacists or others without any limitations.

### Concept

All studies on instruments assessing medication safety competencies of healthcare professionals were considered. Only studies which focused on quantitative self-assessment instruments, scales, questionnaires or surveys that assess healthcare professionals’ medication safety competence overall or that assess safe medication administrations skills/competencies were considered. Instruments that focused on only one specific skill/competency, such as medication calculation skills, were not included as they do not reflect the multidimensional nature of medication safety.

### Context

Studies with participants from any educational, clinical or geographical setting were considered.

### Types of Sources

Due to the nature of the scoping review, where critical appraisal of studies is not intended,^29^,^30^ all types of studies were included – regardless of whether they were peer reviewed. All studies that describe the development of an instrument assessing the medication safety competencies of healthcare professionals (including psychometric testing) or the administration of an instrument were considered. The scoping review included all quantitative or mixed-methods study designs. Qualitative study designs were not included as the scoping review aimed to identify instruments to assess, not describe, the concept of medication safety competence. In order to capture evidence internationally, sources in all languages were included in the screening process if an English or German abstract was available, but data were only extracted if an English or German translation of the full text was available.

## Methods

This scoping review was conducted in accordance with the JBI methodology for scoping reviews,^28^,^31^ and in line with the Preferred Reporting Items for Systematic Reviews and Meta-Analyses extension for Scoping Reviews (PRISMA-ScR).^32^ The completed PRISMA-ScR Checklist is provided in Appendix 1. The a priori protocol^33^ was registered^34^ at Open Science Framework.

### Deviations from the Protocol

Due to time constraints, one person (CS) performed the data extraction and another (DK) checked the extracted data to ensure accuracy and completeness.^35^ Since data extraction is an iterative process, the data extraction form was modified along the way.^35^ Modifications and rationale are described in the data extraction section. In order to obtain a better overview of the content of the dimensions of the MSC represented in the instruments, the data synthesis was modified. The dimensions considered by the instruments were mapped according to the adapted domains of the Safety Competencies Framework.^17^

### Search Strategy

The development of the search strategy was supported and accompanied by a health sciences librarian. A preliminary search of MEDLINE (PubMed) was carried out to identify keywords and index terms used in the titles and abstracts of relevant sources. The keywords and index terms were used to develop the search strategy for each database. Key studies which were identified in a previous manual search were used to validate the search strategy. Search strategies of the different databases were reviewed and approved by the health sciences librarian. MEDLINE (PubMed), CINAHL (EBSCOhost), Web of Science Core Collection, LIVIVO (excl. MEDLINE), Cochrane Database of Systematic Reviews and Cochrane Central Register of Controlled Trials (Ovid) and CareLit were searched in January 2024. An unsystematic search was conducted in CareLit, a German database of German-language nursing journals, as the systematic search functions in this database are limited. The reference lists of all included articles were screened for additional relevant studies.^31^ The search strategy is presented in Appendix 2.

### Study Selection

**Figure 1:**
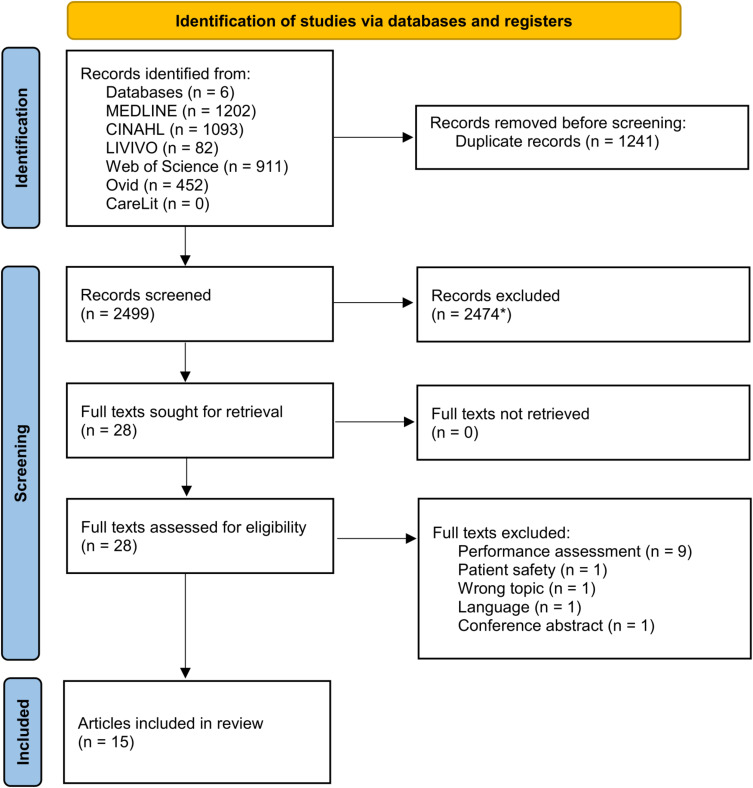
PRISMA flow diagram of the study selection process.

**Notes**: *Subsequently included during data extraction (n = 3). Adapted from Page MJ, McKenzie JE, Bossuyt PM, et al. The PRISMA 2020 statement: an updated guideline for reporting systematic reviews. *BMJ*. 2021:n71.^37^

**Abbreviation**: PRISMA, Preferred Reporting Items for Systematic Reviews and Meta-Analyses.

Citation details of the identified sources were imported into Rayyan,^36^ a web app for systematic reviews. Duplicates were automatically removed by Rayyan if the similarity of the citation details was 95% or higher. For lower similarity, duplicates were removed manually. The entire study selection process was conducted by two independent reviewers (CS, DK) and any disagreements were resolved through discussion or with a third reviewer (CM). The inclusion and exclusion criteria were piloted in title/abstract (n=25) and full text (n=4) screening and subsequently updated in the process. Full texts of included articles were retrieved and imported into Rayyan. Full texts which did not meet the eligibility criteria were excluded and reasons for their exclusion are provided in the PRISMA-Flowchart (Figure 1) and in Appendix 3. For the final analysis, all full texts included were managed using the reference management software Zotero (Corporation for Digital Scholarship, Virginia, USA).

### Data Extraction

The data extraction form developed by the authors (Appendix 4), described in the a priori protocol,^33^ was piloted (n = 3) and modified in the process: The “Concept” category has been specified to extract subscales and item examples. “Subscales” and “Number of items” have been merged for better overview. The “Analysis and Interpretation” and “Development and Measurement Characteristics” categories have been added as they are relevant to finding a suitable instrument. Data were extracted by one researcher (CS), and all were reviewed by another researcher (DK) to ensure accuracy and completeness.^35^

### Data Analysis and Presentation

In order to answer the research question, the extracted data was summarized in tables and analyzed descriptively. Articles included in the synthesis were categorized based on the instruments included (Table 1). The dimensions of MSC considered in the instruments identified were mapped independently by two researchers (CS, VSW) onto the domains of the Safety Competencies Framework^17^ and agreement was reached through discussion (Table 2). As medication safety is an essential component of patient safety,^12^ only minor adjustments in the wording for two of the six original domains were necessary. As a result, the six domains that were used were as follows: Medication Safety Culture; Teamwork; Communication; Safety, Risk and Quality Improvement; Optimize Human and System Factors; Recognize, Respond to and Disclose Medication Incidents. The documented measurement properties were analyzed (Table 3), and the Instruments were analyzed using the PCC framework, including scale rating (Table 4). The primary source of information on instrument content and measurement properties was the original reference of the instrument. As a scoping review was conducted, no critical appraisal of the included articles was performed, nor any risk of bias assessed.^32^

**Table 1:** Articles Included to Data Analysis and Synthesis; in Alphabetical Order, by Instrument

Author	Year	Country	Instruments Identified in Study	Instrument’s Language
**Park & Seomun**^38^	2021	Korea	Medication Safety Competence Scale (MSCS)	English
**Yang et al^39^**	2021	China	MSCS Chinese version	Chinese
**Mohammadi et al^40^**	2023	Iran	MSCS Persian version	Persian
**Mohebi et al^41^**	2024	Iran	MSCS Persian Version	Persian
**Korkut & Ulker^42^**	2023	Turkey	MSCS Turkish Version	Turkish
**Lappalainen et al^43^**	2020	Finland	Medication Safety Scale (MSS)	English
**Mygind et al^44^**	2018	Denmark	N.N. (no name described)	English
**Salcedo-Diego et al^45^**	2017	Spain	NORMA	English
**Salcedo-Diego et al^46^**	2022	Spain	NORMA	English
**Aggar & Dawson^47^**	2014	Australia	Preparedness for oral medication administration questionnaire	English
**Aggar et al^48^**	2018	Australia	Preparedness for oral medication administration questionnaire	English
**Lapkin et al^49^**	2015	Australia	Theory of Planned Behaviour Medication Safety Questionnaire (TPB-MSQ)	English
**Omura et al^50^**	2015	Japan	TPB-MSQ Japanese Version	Japanese
**Secginli et al^51^**	2021	Turkey	TPB-MSQ Turkish Version	Turkish
**Fernandez et al^52^**	2023	Australia; South Africa; India, Turkey	TPB-MSQ TPB-MSQ Turkish Version	English Turkish

**Table 2:** Instruments’ MSC Dimensions Mapped to the Adapted Form of the Safety Competencies Framework

MSC Domains (adapted from Safety Competencies Framework^17^)	MSCS^38^	MSS^43^	N.N.^44^	NORMA^45^	Preparedness for oral Medication administration Questionnaire^47^	TPB-MSQ^49^
**Medication Safety Culture**	x	x	x			(x)*
**Teamwork**	x	x	x		(x)	(x)*
**Communication**	x	x	x		x	
**Safety, Risk and Quality Improvement**	x	(x)			x	
**Optimize Human and System Factors**	x	(x)				
**Recognize, Respond to and Disclose Medication Incidents**	x	x	(x)	x	x	(x)*

**Notes**: (X)=partially/in outline; *=assumed based on the descriptions in the text.

**Table 3:** Measurement and Psychometric Properties of the Identified Instruments; in Alphabetical Order

Instrument	Item Creation		Validity					Reliability	
	Item Creation/ Derived from	Face / Content validity	Pilot Testing	Sample size	Item retention	Construct Validity		Internal Consistency	Test-retest
						Factorability	Exploratory (EFA) and Confirmatory (CFA) factor analysis		
**MSCS^38^**	Literature review and in-depth interviews	Item-level content validity index (I-CVI)=0,7–1.0 4 items removed (cut-off=0.8)	Tested by nurses (n=6)	n=607	Items selected with interitem correlation ≥0.4	Barlett’s Test of Sphericity analysis= significant data Kaiser-Meyer-Olkin (KMO)=0.953	EFA (n=300) revealed six factors explained 63.2% of the total variance Kaisers criterion -> items with eigenvalue ≥ 1.0 and factor loading ≥ 0.40 were selected CFA (n=307) χ2=1847.27; df=579; RMR=0.037; RMSEA=0.085; CFI=0.825; GFI=0.743; IFI=0.826; TLI=0.809; AVE=0.628–0.763; Composite reliability=0.874–0.958	Cronbach’s alpha=0.96	n=120 ICC=0.78
**MSS^43^**	Literature review		Tested by nursing science students (n=12)	n=161				Cronbach’s alpha for the individual subscales = 0.724–0.864	
**N.N.^44^**	Learning objectives of the educational program		Pilot resulted in changing words	n=75					
**NORMA^45^**	Adaptation of Irujo et al’ questionnaire^53^ and literature review	Items were rated on pertinence and with Position Index (cut-off 0.70); Discussion and reduction of items in an expert panel	Tested by nurses (n=17)						
**Preparedness for oral medication administration questionnaire**^47^	Adaptation of Fisher & Parolins’ questionnaire^54^			n=88				Cronbach’s alpha=0.89	
**TPB-MSQ**^49^	Literature review and focus group interviews	Expert panel review and item rating for accuracy, clarity and ability to measure what was intended= agreed on 96% Scenarios where adjusted and 12 items deleted		n=65				Cronbach’s alpha=0.844	

**Abbreviations**: RMR, root mean square residual; RMSEA, root mean square error of approximation; CFI, comparative fit index; GFI, goodness-of-fit index; IFI, incremental fit index; TLI, Tucker-Lewis index; AVE, average variance extracted; ICC, intraclass correlation coefficient.

**Table 4:** Characteristics of the Identified Instruments; in Alphabetical Order

Instrument	Targeted HCPs (Participants) Developed for (D)/ Applied in (A)	Setting (Context) Developed for (D)/ Applied in (A)	Total number of Items; Number of Items Per Subscale (Concept)	Scale type	Analysis and Interpretation
**Medication Safety Competence Scale (MSCS)^38^**	D: Nurses A: Nursing students^41^	D: Clinical setting A: Educational setting^41^	N = 36; Patient-centered medication management (n = 9) Improvement of safety problems (n = 8); Management of effecting factors (6); Safety risk management (n = 6); Multidisciplinary collaboration (n = 4); Responsibility in the nursing profession (n = 3)	5-point Likert scale	Sum score: Ranges from 36 to 180 Interpretation of sum score:^40^ 36–75 = poor 76-130 = moderate 131–180 = favorable MSC
**Medication Safety Scale (MSS)^43^**	D: Nurses	D: Clinical setting	N = 45; Working conditions factors (n = 13); Individual factors (n = 12); Systemic factors (n = 9); Drug administration n = (9); Knowledge of medicines (n = 2)	5-point Likert scale	Total mean score (ranges from 1–5) Subscale mean score (range from 1–5) Interpretation: 1 = low medication safety in the hospital unit 5 = high medication safety in the hospital unit
**NORMA^45^**	D: Nurses A: Midwives^46^	D: Clinical Setting	N = 45 (bundled in 34 questions); Attitude (n = 20) Knowledge (n = 12) Skill (n = 13)	5-point Likert scale	Sum score: Ranges from 34 to 170 Interpretation:^46^ 34 = low overall MIs reporting competence (OC) 170 = high OC; Weighted subscale scores (range from 11 to 57) Interpretation:^46^ 11 = low subscale score 57 = high subscale score
**Preparedness for oral medication administration questionnaire^47^**	D: Nursing students	D: Educational setting	N = 17; No subscales described	6-point Likert scale	Sum score: Ranges from 17 to 102 Interpretation: 17 = low total preparedness score 102 = high total preparedness score
**Theory of Planned Behaviour Medication Safety Questionnaire (TPB-MSQ)^49^**	D: Nursing, pharmacy and medicine students A: Nurses^50^,^52^	D: Educational setting A: Clinical setting^50^,^52^	N = 49; Attitude (n = 15); Perceived behavioural Control (n = 14); Subjective norms (n = 12); Behavioural intentions (n = 4); Decision difficulty (n = 4)	7-point Likert scale and yes/no-questions	Subscale mean score (ranges from 1 to 7) Interpretation: 1 = low intention to behave in a manner that promotes medication safety 7 = high intention to behave in a manner that promotes medication safety^52^ Analysis of subscales behavioural intentions and decision difficulty not described
**N.N.^44^**	D: Social worker; social and healthcare assistants, social and healthcare helpers, non-healthcare background, manager, nurses	Clinical setting	Unknown, minimum 22; Role clarification (n = 4); Patient safety culture (n = 8); Medication handling (n = 5); Patient empowerment (n = 3); Communication with healthcare professionals (2)	Different 5-point Likert scales	Dichotomized analysis of each item: eg (“to a very high degree” + “to a high degree”) = positive outcome (“to some degree” + “to a lower degree” + “to a much lower degree”) = negative outcome Evaluation of patient safety awareness (not reported in the article) via responses to at-risk situations

## Results

### Study Inclusion

The search in the databases yielded 3740 results. 1241 duplicates were removed and 2499 titles and abstracts were screened, of which 25 sources were eligible for full text screening. 13 were excluded as they did not meet the eligibility criteria. Three articles found through citations of included articles during data extraction were subsequently included, resulting in 15 articles included in this review.^38–52^

The descriptions of the included articles according to the data extraction form are available in Appendix 5. The PRISMA flowchart (Figure 1) shows the study selection process.

### Characteristics of Included Sources of Evidence

The included articles were published between 2014 and 2024 and were from twelve different countries, including four from Australia,^47–49^,^52^ two each from Iran,^40^,^41^ Spain^45^,^46^ and Turkey^42^,^51^ and one each from China,^39^ Denmark,^44^ Finland,^43^ Korea,^38^ and Japan.^50^ All sources were scholarly publications in journals and primary research, reviews were not included. Most of the articles describe the development, translation or adaptation of instruments (n = 11).^38–40^,^42–45^,^47^,^49–51^ The other articles exclusively focus on the application of instruments (n = 4).^41^,^46^,^48^,^52^

### Review Findings

Six instruments were identified from the 15 articles included (Table 1). All instruments were originally developed and published in English. Two of the instruments were translated into other languages: The Medication Safety Competence Scale (MSCS)^38^ has been translated into Chinese,^39^ Turkish^42^ and Persian.^40^ The Theory of Planned Behaviour Medication Safety Questionnaire (TPB-MSQ)^49^ has been translated into Japanese^50^ and Turkish.^51^

Two instruments^38^,^43^ could be mapped to all six MSC domains of the adapted Safety Competencies Framework (Table 2). The content of one instrument^45^ could only be assigned to one domain, “Recognize, Respond to and Disclose medication incidents”. At the same time, this domain is the only one matching all instruments. “Teamwork” could be assigned to five of the six instruments, “Optimize Human and Systemic Factors” to the fewest instruments (n = 2).

The items of four of the six instruments were derived from the literature (Table 3). Additionally, interviews were conducted to develop the items for the MSCS and focus group interviews for the TPB-MSQ. For the development of the NORMA and the preparedness for oral medication administration questionnaire, Irujo et al’ questionnaire^53^ and Fisher & Parolins’ assessment tool^54^ were adapted, respectively. N.N.’s items reflect the learning objectives of the educational program for which the instrument was designed.

Psychometric properties: Sample sizes ranged from 65 to 607, with no description of calculation methods. NORMA^45^ was only described up to the pilot test. Cronbach’s alpha of the instruments (n = 3)^38^,^47^,^49^ ranged from 0.844 to 0.96, with only one article^43^ reporting the Cronbach’s alpha for the individual subscales (0.724–0.864). Face validity was reported in three instruments,^38^,^45^,^49^ and item-level content validity index was reported in one instrument. 38 Pilot testing (n = 4)^38^,^43–45^ was conducted with 6–17 participants, with one study^44^ not reporting a number. Statistical item retention, factorability tests, exploratory factor analysis and confirmatory factor analysis were performed for one instrument.^38^ One instrument described test–retest reliability.^38^

All six identified instruments were developed for nurses or nursing students (Table 4). Two instruments were developed for more than one target group. The TPB-MSQ^49^ was developed for nursing, pharmacy and medicine students and the N.N.^44^ was developed to assess the competence of social worker, social and healthcare assistants/helpers, non-healthcare background manager and, only one healthcare profession, nurses. Other than nurses, the NORMA was administered on to midwives.^46^

All instruments were designed in or for the clinical setting, except for the Preparedness for oral medication administration questionnaire^47^ and the TPB-MSQ.^49^ Five of the six instruments were used in clinical settings, as the application of the TPB-MSQ in clinical setting was described.^50^,^52^ The application of the MSCS in educational settings has also been described.^41^ Thus, three of the six instruments^38^,^47^,^49^ were applied in educational settings. All instruments use 5- to 7-point Likert scales as rating method (Table 4). The number of items ranges from 17 to 49. For the N.N.^44^ no final number of items is described. Based on the explanations in the text, it can be assumed that there are at least 22 items. For the TPB-MSQ^49^ 56 items including 5 questions on demographics are described, but the item breakdown shows only 54 items including demographics. Five of the six instruments have subscales.^38^,^43–45^,^49^ The scales differ in the way they are scored. Three instruments^43^,^45^,^49^ allow for a specific analysis and interpretation of subscales.

## Discussion

The aim of the scoping review was to identify instruments to measure healthcare professionals’ medication safety competence. Six different instruments were identified in 15 included articles. Two instruments^38^,^49^ were translated from English into two^50^,^51^ or three^39^,^40^,^42^ other languages.

With the MSCS and the MSS being at least partially assigned to all MSC domains, two instruments could be identified that represent the multidimensionality of the MSC. The fact that the MSC domain “Recognize, Respond to and Disclose Medication Incidents” is covered by all instruments, is in line with the central task of promoting medication safety, as formulated in the WHO’s Global Patient Safety Challenge “Medication Without Harm”: To reduce preventable medication-related harm by addressing harm resulting from errors and unsafe practices.^55^ “Teamwork” as the second most assigned domain may indicate, that interprofessional collaboration is key to promoting medication safety. The domain “Optimize Human and System Factors” could only be found in two instruments, which may suggest, that it is the area of least awareness or, perhaps that it is not considered possible to change them.

Even though several publications on the development and evaluation of instruments exist,^56–59^ instrument development, validity and reliability testing was described scarcely and only one article^38^ described the development and validation process comprehensively. Face and content validity are very important to ensure that the items measure what they are supposed to and are comprehensive.^56^,^60^ The Preparedness for oral medication administration questionnaire and the TPB-MSQ were not pilot tested, which may have resulted in poorly worded and unnecessary items.^56^ The number of participants in the described pilot tests was rather small, although recommendations vary.^56^,^61^ Construct validity tests were only performed for the MSCS and the sample size of 607 was evaluated good.^38^ According to present discussion, the sample sizes of the studies of the other instruments do not appear to be sufficient for further statistical evaluation.^62^ The measurement properties of the MSCS do not fully meet the criteria for good measurement properties according to the COSMIN guideline.^60^ The internal consistency of the MSCS,^38^ the MSS,^43^ the Preparedness for oral medication administration questionnaire^47^ and the TPB-MSQ^49^ can be rated as good.^56^,^60^

Descriptions of the development and application of the instruments show that five of the six instruments are applicable in clinical settings^38^,^43–45^,^49^ and three in educational settings.^38^,^47^,^49^ Instruments were mostly developed for the nursing profession. Despite the essential importance of physicians and pharmacists in the medication process and in ensuring medication safety, no instrument could be identified that was developed to assess their competence. Only the TPB-MSQ^49^ was used to also survey medical and pharmacy students. Interprofessional collaboration is a powerful concept for promoting and ensuring medication safety, including through a better coordination of HCPs’ therapeutic approaches and optimized care processes.^63–65^ Thus, instruments are needed that can be used in various healthcare professions including physicians and pharmacists. Particularly in the care of polypharmacy patients, all healthcare professions involved need to be competent in medication safety. Only then can MEs be prevented, processes like de-prescribing initiated and safety ensured. At the same time, the result reinforces the important role attributed to nurses in the medication process.^66^

## Limitations

Due to the scoping review methodology, without performance of critical appraisal^29^,^30^ of the studies included and differences in the way data were reported in the articles, results and comparisons of results should be treated with caution and no direct conclusions should be drawn from them. A systematic and exhaustive search was conducted, but only articles written in English and German were included, which limits the applicability of the review results to the English and German-speaking world. The validity of the results could have been increased if the entire data extraction process had been carried out by two independent researchers and if authors of the instruments included would have been contacted.

## Conclusions

The scoping review identified six instruments^38^,^43–45^,^47^,^49^ measuring healthcare professionals’ MSC. Two instruments^38^,^43^ cover the multidimensionality of MSC by reflecting all the six domains. Since only the MSCS^38^ has undergone extensive psychometric evaluation and the results can be considered as acceptable, the MSCS can be used to assess MSC. The MSCS has been translated into three other languages,^39^,^40^,^42^ making it available to a wider audience enabling comparison of results in other settings and countries. However, the MSCS was developed and validated in nurses. No instruments to measure MSC were identified for other healthcare professionals.

## Implications for Research

In order to further improve medication safety in a targeted manner, instruments are needed to measure the MSC of all healthcare professionals. This can be achieved, either by adapting and evaluating existing instruments, such as the MSCS, or by developing new instruments. Suitable instruments need to be translated into other languages and psychometrically evaluated in order to assess MSC and to compare results with other health professions and settings. The assessment of medication safety competence and identification of areas in which healthcare professionals need improvement in MSC could then lead to interventions to improve medication safety competence and hence medication safety.

## Data Availability

Data supporting the results are shown in the Appendices. Other data are available upon request from the corresponding author.
